# Placental biology links genetic, epigenetic, ancestral, and social determinants to maternal–fetal health inequities

**DOI:** 10.3389/frph.2026.1783837

**Published:** 2026-04-15

**Authors:** Viviane Schuch, Indrani Mukherjee, Alan Rodrigo Nascimento de Carvalho, Raimot Olanrewaju, Jerrica Davis, Shakera Thomas, Rana Chakraborty, Erica L. Johnson

**Affiliations:** 1 Department of Microbiology, Biochemistry, and Immunology, Morehouse School of Medicine, Atlanta, GA, United States; 2 Division of Pediatric Infectious Diseases, Department of Pediatrics, Miller School of Medicine, University of Miami, Miami, FL, United States; 3 Santo Amaro Campus, Centro Universitario Senac, Sao Paulo, Brazil; 4 Department of Obstetrics and Gynecology, Morehouse School of Medicine, Atlanta, GA, United States

**Keywords:** epigenetics, genetic ancestry, maternal-fetal health, placenta, pregnancy complications, social determinants of health

## Abstract

Black and African American women in the United States experience disproportionately high rates of maternal and infant mortality and morbidity, including placenta-mediated complications such as preeclampsia, preterm birth, and miscarriage. These inequities arise from the interaction of social and environmental conditions with biological pathways that become embedded over the life course, with the placenta serving as a central mediator. In this review, we examine how placental biology integrates genetic variation, epigenetic regulation, paternal contributions, immune processes, and lived social context to shape pregnancy outcomes. We synthesize evidence showing that placental dysfunction, particularly immune dysregulation driven by infectious, environmental, and psychosocial stressors, contributes to placenta-mediated complications with disproportionate impacts among African American women. We highlight how reliance on racial categories in U.S. biomedical research can obscure biological inference and argue for ancestry-informed approaches, alongside self-identified ethnicity, to disentangle inherited genetic variation from socially patterned exposures. Surveying published genome-wide association studies, we show that underrepresentation of African-ancestry populations remains a major constraint on discovery and translation in pregnancy genomic research. We further describe how placental epigenetic mechanisms link socioeconomic and environmental exposures to fetal development, placental aging, and long-term maternal–child health. Finally, we discuss how pregnancy and placental datasets remain sparse and fragmented, and consider how machine-learning approaches may improve pregnancy risk prediction when designed with equity in mind, by integrating placental multi-omics, ancestry-aware genomics, clinical data, and social determinants. Together, this review positions the placenta as a critical interface through which structural inequities shape maternal–fetal health and identifies priorities for more inclusive and equitable research and health care.

## 1 Introduction

**Figure 1:**
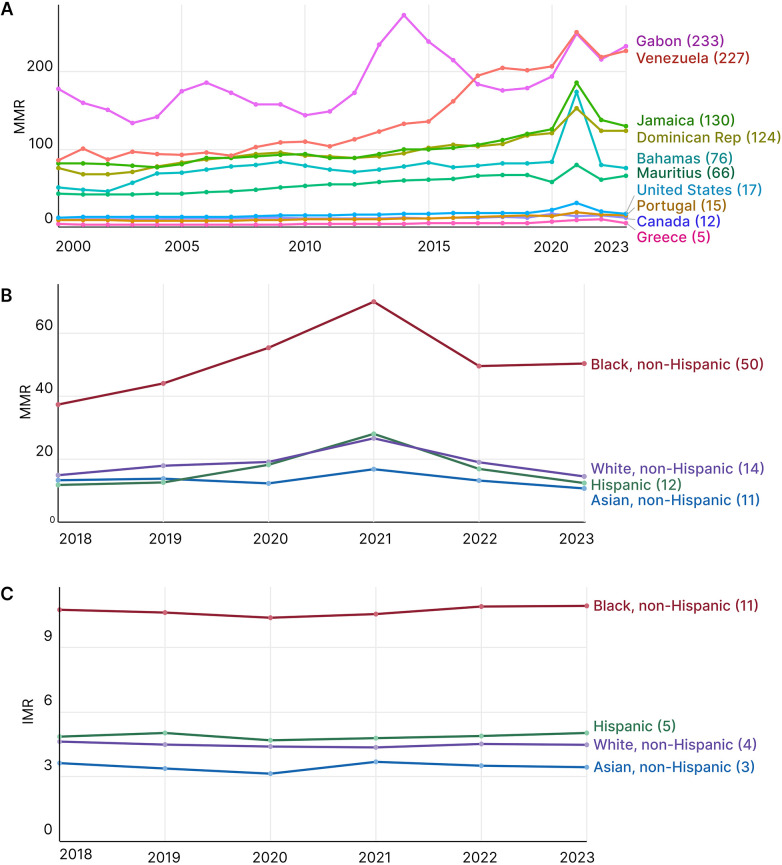
Patterns of maternal and infant mortality. **(A)** Annual maternal mortality ratio (MMR) from 2000 to 2023. The 10 countries with the most significant percentage increase are shown. Values for 2023 are indicated on the right. Source: WHO/UNICEF/UNFPA/World Bank/UN DESA MMEIG (Revised 2025). **(B)** United States MMR stratified by race and ethnicity (2018–2023). Values for 2023 are indicated on the right. Source: NCHS, National Vital Statistics System, Maternal Mortality Statistics. **(C)** United States infant mortality rate (IMR) stratified by race and ethnicity (2018–2023). Values for 2023 are indicated on the right. Source: NCHS, National Vital Statistics System (linked birth/infant death file), 2023. MMR, maternal deaths per 100,000 live births; IMR, infant deaths per 1,000 live births. The WHO series is model-based and subject to periodic revision.

Maternal mortality in the United States (U.S.) remains high compared with other high-income countries and is unequally distributed across populations, with Black/African American women bearing the highest burden (Figures 1A,B) (1, 2). While many countries have reduced the maternal mortality ratio (MMR; deaths per 100,000 live births) over the past 2 decades, the U.S. experienced the seventh-largest percentage increase in MMR globally from 2000 to 2023 (Figure 1A) (1). In the U.S., the MMR rose from 17.4 in 2018 to 32.9 in 2021, then declined to 19.0 in 2023 (2). However, this improvement has not been shared equally. In 2023, African American women experienced 50.3 deaths per 100,000 live births, compared to 14.5 among non-Hispanic White women and 12.4 among Hispanic women. This disparity was even more pronounced in 2021, when the MMR for African American women approached 70 deaths per 100,000 live births, more than twice the national average (Figure 1B) (2).

These health inequities reflect the intersection of social determinants and biological pathways, in which the placenta, the dynamic fetal organ at the maternal-fetal interface, plays a central mechanistic role. A greater burden of placenta-mediated complications, including preeclampsia, preterm birth, gestational diabetes, and miscarriage, contributes substantially to adverse maternal and infant outcomes in African American populations (3–6) and is associated with infant mortality rates that are more than double those of White/European American infants (Figure 1C) (7).

**Figure 2:**
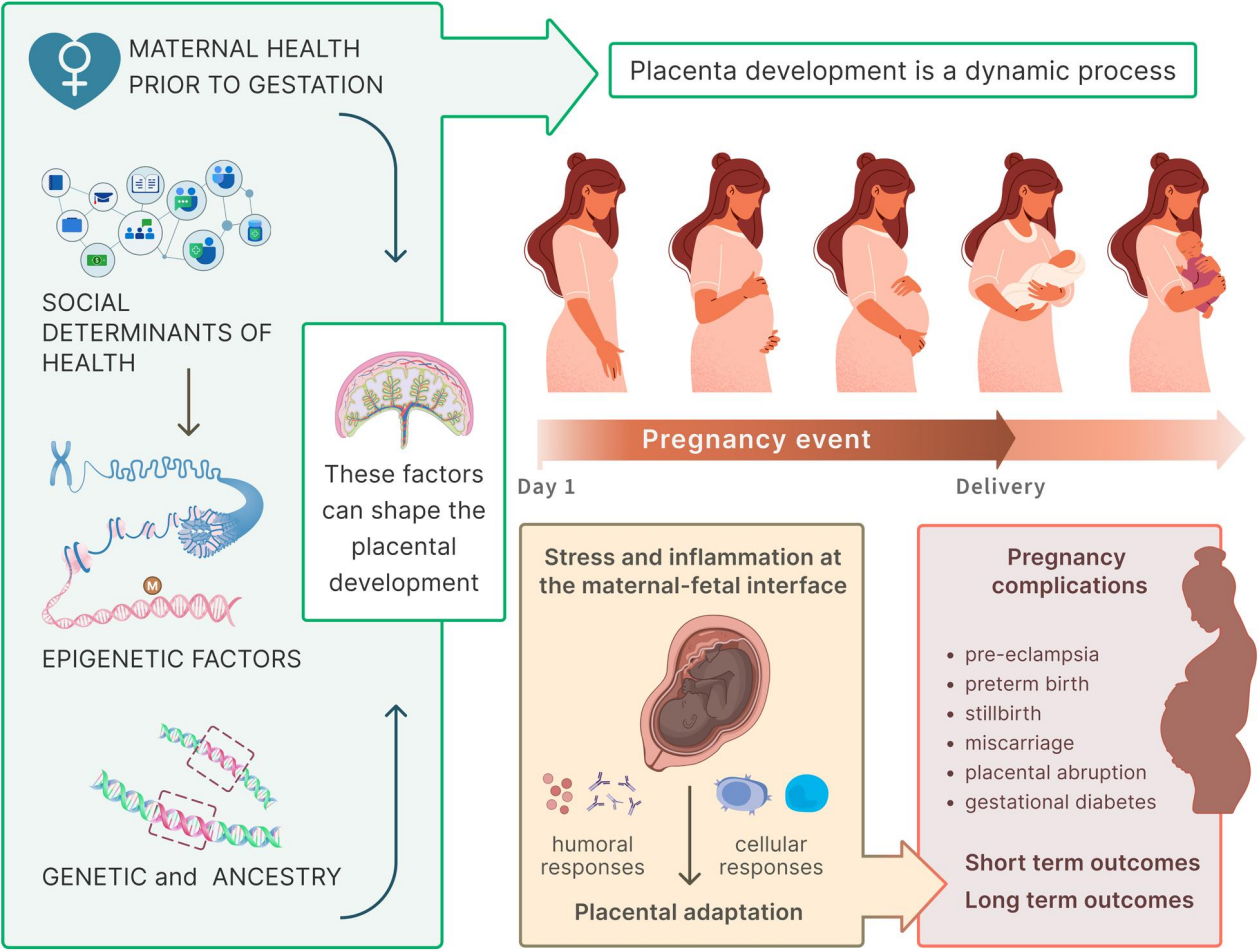
Interplay of maternal, genetic, epigenetic, and social factors shaping placental development and pregnancy outcomes. The figure highlights the placenta as the central mediator at the maternal–fetal interface. Preconception health, social determinants of health, genetic and ancestry-related variation, and epigenetic modifications collectively shape pregnancy trajectories. Disruptions in placental function (e.g., inflammatory activation and/or stress) can drive placental adaptations and complications (preeclampsia, preterm birth, stillbirth, miscarriage, placental abruption, and gestational diabetes) with short- and long-term consequences for maternal and child health.

Pregnancy outcomes result from a combination of maternal health, genetic factors, social determinants of health, paternal contributions and epigenetic regulation (Figure 2) (8–12). The placenta integrates these signals and adapts throughout gestation to regulate the maternal–fetal interface. Disruptions in placental function, including inflammation and oxidative stress, may trigger maladaptive responses that drive pregnancy complications and compromise both short-term and long-term outcomes for mothers and children (13).

This paper aimed to review placental biology, focusing on how genetic variation, social context, and epigenetic modulation influence maternal–fetal outcomes. We highlight key conceptual advances, identify gaps in current research, and discuss how these insights can guide equity-focused precision medicine.

## 2 From historical injustices to genomic innovations: addressing maternal health inequities

The persistent health inequities experienced by pregnant African American women in the U.S. reflect the long-term effects of policies, practices, and structural racism. In the early twentieth century, eugenic theories justified policies enforcing coercive sterilization and severely restricted the reproductive rights of countless African American women under the guise of “public health”. These polices were justified by biased and racist claims of genetic inferiority (14). The legacy of eugenics has shaped the structural racism that still permeates U.S. healthcare delivery (15, 16).

By the mid-20th century, researchers increasingly recognized the role of social determinants of health (SDoH), such as employment and lifestyle, in influencing health outcomes at the individual and population levels (17). The Civil Rights Movement of the 1950s and 1960s heightened national awareness of inequities in all areas of U.S. society, including unequal access to and treatment within the healthcare system for African Americans (18). This period marked a significant shift from genetic determinism to a broader understanding of the drivers of disease and health.

The biopsychosocial model emerged during the latter half of the 20th century. According to this model, health outcomes result from the interaction among biological, psychological, and social factors. This perspective is consistent with current concepts on syndemics and social determinants of health (19). Concurrently, the Women's Health Movement advocated for a more patient-centered and culturally sensitive healthcare system (20). As the 20th century ended and the 21st century began, attention increasingly focused on how institutional structures and historical patterns contributed to persistent health inequities across U.S. populations (21).

More recent maternal health research has incorporated genetic, epigenetic and precision medicine approaches to better understand biological pathways involved in pregnancy complications. Genetic ancestry research has identified variants that differ in frequency across populations and may influence susceptibility to certain pregnancy-related disorders (22). For example, APOL1 G1 and G2 risk alleles, which are enriched in individuals of African ancestry, have been associated with increased risk of preeclampsia and kidney disease (23, 24).

Epigenetic research has provided mechanistic insight into how social determinants of health, including chronic stress, discrimination, and socioeconomic adversity, may become biologically embedded during pregnancy (11, 25–27). Recent epigenome-wide studies have identified differential DNA methylation patterns in placental and maternal tissues associated with socioeconomic disadvantage and pregnancy complications in cohorts that include large proportions of African American women. For example, analyses from the ELGAN cohort demonstrated that maternal socioeconomic adversity is associated with altered placental DNA methylation in genes involved in immune signaling, transcriptional regulation, and stress-response pathways (28). Similarly, epigenetic studies of preterm birth in African American populations have identified methylation differences in genes related to inflammatory, metabolic, and cardiovascular pathways that may contribute to intergenerational risk (29).

Building on these advances, the integration of large-scale datasets and advanced analytical approaches has enabled the development of multivariable risk prediction models for pregnancy complications (30). A widely recognized example is the Fetal Medicine Foundation's first-trimester screening algorithm for preeclampsia, which integrates maternal characteristics, blood pressure, uterine artery Doppler measurements, and placental biomarkers to identify pregnancies at elevated risk (31). In the ASPRE trial, this strategy enabled targeted low-dose aspirin prophylaxis and significantly reduced the incidence of preterm preeclampsia (32).

Regardless of these scientific and technological advances, improvements in pregnancy outcomes have not been evenly distributed. African American women in the United States continue to experience disproportionately high rates of pregnancy-related morbidity and mortality. Understanding why genomic innovation has not yet translated into proportional improvements for these populations remains an urgent priority for maternal-fetal health research. In the following sections, we examine key structural and scientific gaps that limit progress and outline strategies to address them.

## 3 The placental data gap and the need for diversity

The placenta serves as a critical interface in fetal development and maternal health, mediating biological mechanisms that contribute to maternal-fetal health inequities. Understanding placental biology is therefore essential for addressing these disparities. However, studying the placenta poses unique challenges due to its complexity as a multifunctional, transient organ. Despite its central role, the placenta remains historically understudied and underrepresented in primary genomic resources, such as the Genotype-Tissue Expression (GTEx) project (33, 34). This gap limits our understanding of placental genomics and restricts the development and validation of placenta-specific computational tools. Clinical data regarding pregnancy are also sensitive, fragmented, and often inconsistently captured across health systems and biobanks (35, 36). Pregnancy episodes are identified using multiple EHR (electronic health record) domains, and key elements such as gestational age, delivery timing, and outcomes might be missing or unavailable due to privacy concerns (36, 37). In addition, paternal data are rarely linked to placental datasets, limiting the ability to model dyadic contributions and parent-of-origin mechanisms in placenta research (38, 39).

Beyond the placental data gap, another inequity is the underrepresentation of diverse populations in human genomics. Decades of Eurocentric sampling mean that historically marginalized groups, especially people of African descent, are sparsely represented in discovery and validation cohorts. In 2025, the GWAS (Genome-wide association study) Diversity Monitor reported that African American and Afro-Caribbean participants comprised 3.7% of GWAS samples (about 4% when combined with African-ancestry participants), while 87.8% of European participants were included (40, 41). Participants of African descent are also underrepresented in clinical trials and longitudinal cohorts (42, 43). Correcting this imbalance is critical for ensuring maternal health equity. An equity-first strategy, developed in partnership with communities, that diversifies the genomics workforce, embeds ancestry and social context, and moves beyond race as a biological concept might produce results that are more generalizable and transferable across populations (44).

Large-scale resources are beginning to close long-standing gaps in sampling and reference design. The All of Us Research Program is enrolling more than one million U.S. participants and links genetic, clinical, environmental, and lifestyle data longitudinally, enabling improved risk profiling and hypothesis generation across populations, including African American participants and other underrepresented minorities (45, 46). Region-focused efforts, such as the African Genome Variation Project, have mapped variation across multiple African populations, providing essential context for allele frequencies and haplotypes (47).

At the reference level, GRCh38, a linear composite assembled from a limited donor set, left approximately 8% of the genome unresolved, particularly in repetitive and centromeric regions, and encoded a single haplotype per locus (48). These design constraints affect variant discovery, gene–disease association studies, and downstream analyses. The telomere-to-telomere (T2T-CHM13) assembly closes those gaps and corrects structural errors, whereas the human pangenome reference provides a graph-based, multihaplotype representation built from many genomes (48, 49). These alternative references improve read alignment and variant detection, including insertions, deletions, and rearrangements across the breadth of human genetic variation.

Without a concerted effort to dismantle historical inequities in medical research, advancements in maternal-fetal biology will continue to perpetuate, rather than eliminate, health inequities. A paradigm shift toward genomics-informed, equity-focused care is urgently needed, one that combines cutting-edge science with a strong commitment to health equity. Central to this effort is the placenta, the organ that integrates biological and environmental signals during pregnancy.

## 4 The placenta and its role in maternal-fetal health

As a complex organ exclusive to eutherian mammals, the placenta has enabled prolonged gestation and advanced fetal development, offering reproductive advantages that have driven mammalian success and diversification across evolution (50, 51). The placenta transfers nutrients and oxygen from the mother to the fetus while removing waste products, thereby supporting the increasing metabolic demands and complex development (52, 53).

**Figure 3:**
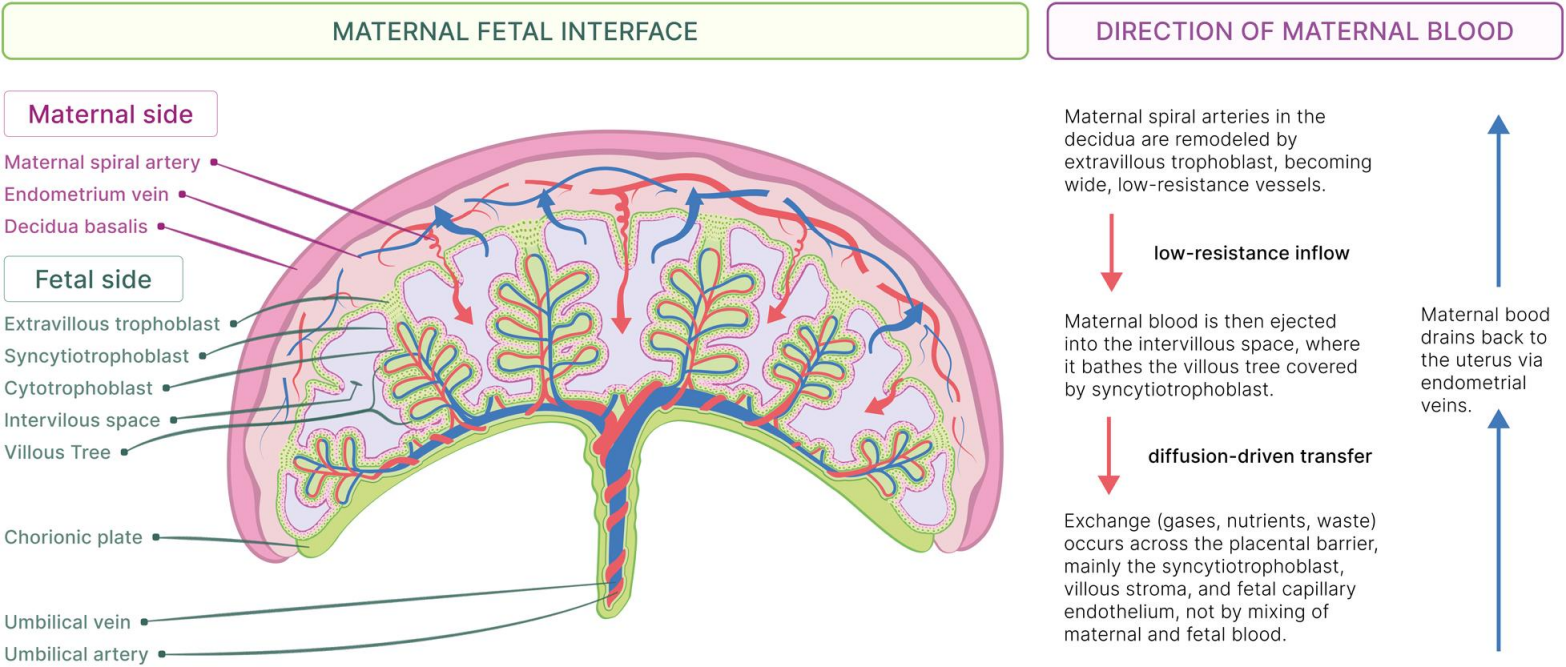
Maternal–fetal interface and direction of maternal blood flow. Schematic cross-section of the human placenta showing the maternal side and fetal side. Extravillous trophoblasts remodel maternal spiral arteries in the decidua basalis into wide, low-resistance vessels that deliver maternal blood into the intervillous space. The maternal blood bathes the villous tree, which is covered by syncytiotrophoblasts, where exchange occurs across the placental barrier without mixing of maternal and fetal blood. Maternal blood returns to the uterus via the endometrial vein, and fetal blood circulates through the umbilical arteries and the umbilical vein.

This process begins when maternal blood is delivered to the intervillous space on the fetal side via remodeled spiral arteries, where it bathes the villous tree covered by the syncytiotrophoblast layer. Exchange of gases, nutrients, and wastes occurs across the placental barrier (syncytiotrophoblast, cytotrophoblast, villous stroma, and fetal capillary endothelium) via diffusion, facilitated diffusion and active transport (Figure 3). In humans, the role of the placenta is vital because of the extended period of fetal brain development, leading to significant cognitive and neurological benefits (53).

In addition to its primary functions, the placenta orchestrates essential processes that sustain a healthy pregnancy. These processes are not static but are finely tuned to meet the dynamic needs of the developing fetus and adapt to changes in maternal metabolism across gestation. As a hormone-producing organ, the placenta synthesizes and secretes hormones such as human chorionic gonadotropin (hCG), progesterone, estrogen, placental lactogen, melatonin, corticotropin-releasing hormone, and placental growth factor, all of which are vital for maintaining pregnancy and supporting fetal growth (54, 55). Additionally, it acts as a selective barrier, regulating the passage of nutrients, gases, and waste while protecting the fetus from harmful substances and pathogens (56).

The placenta is also an immunological organ that must balance two competing demands: establishing tolerance to a semi-allogenic (HLA-discordant) fetus while maintaining defenses that protect the fetus from infection. This balance is achieved through coordinated mechanisms at the maternal–fetal interface. Trophoblast cells limit classical HLA expression while expressing nonclassical molecules such as HLA-G, which interact with maternal immune receptors to reduce cytotoxic responses. In parallel, specialized immune populations in the decidua, including decidual natural killer cells, decidual macrophages, and regulatory T cells, support tissue remodeling and promote maternal immune tolerance. At the same time, trophoblasts maintain intrinsic innate immune defenses, including pattern-recognition receptor signaling and antiviral responses, that help restrict pathogen invasion and vertical transmission while preserving fetal tolerance (57).

Moreover, the placenta releases neurotransmitters such as serotonin, dopamine, and epinephrine/norepinephrine, which are critical for regulating fetal brain development and may be the primary source of these neurotransmitters during early brain formation (58). These processes are intricately coordinated to align with the varying requirements of each stage of fetal development, from the establishment of early pregnancy to the preparation for birth.

The developmental origins of health and disease (DOHaD) model, proposed by David Barker, provides robust evidence that the foundations of adult health, including cardiometabolic risks, are shaped by social determinants of health and environmental exposures during fetal and early life (59, 60). The DOHaD model emphasized the placenta's role in these developmental processes. Adverse SDoH, such as poor maternal nutrition or chronic stress, can lead to compensatory placental adaptations that may predispose the fetus to health issues in adulthood (61). Importantly, DOHaD research has historically centered on maternal pregnancy exposures, which can obscure the contribution of paternal and broader family influences. Addressing this imbalance is increasingly recognized as necessary for stronger causal inference and more complete models of intergenerational risk (38). This framework is particularly relevant for populations facing a higher burden of cardiometabolic disease, chronic stress, and structural inequities, including higher prevalence of hypertension, obesity, environmental pollutant exposure, and barriers to consistent prenatal care, conditions that increase the risk of placental dysfunction and related pregnancy complications in the U.S., including among African American women (62).

Despite its critical functions, systematic analysis of placental tissues remains limited, even though the placenta is the only human organ fully available for biospecimen collection and pathological examination after pregnancy ends (63). This gap reflects both the biological complexity of the placenta and its historical underrepresentation in biomedical research, which has contributed to major gaps in our understanding of placental biology and disease mechanisms (56). Indeed, most studies have examined maternal and fetal health separately, often overlooking the placenta as the central biological interface between them. This gap has contributed to limited mechanistic understanding and fewer targeted interventions for placental-specific disorders (64, 65). Among interventions, early delivery is a primary medical approach for several placental complications, such as preeclampsia, despite the risks associated with prematurity (66). Although recent advancements in placental imaging and molecular profiling are improving detection and mechanistic understanding of placental dysfunction, developing preventive treatments for placental disorders remains challenging (67). Current medical practices tend to manage rather than prevent placental disorders, reflecting limitations in early detection markers (68). Therefore, research and clinical practice focused on placental health are urgently needed to address these challenges.

## 5 Placental immunity and inflammation: critical drivers of adverse pregnancy outcomes

**Figure 4:**
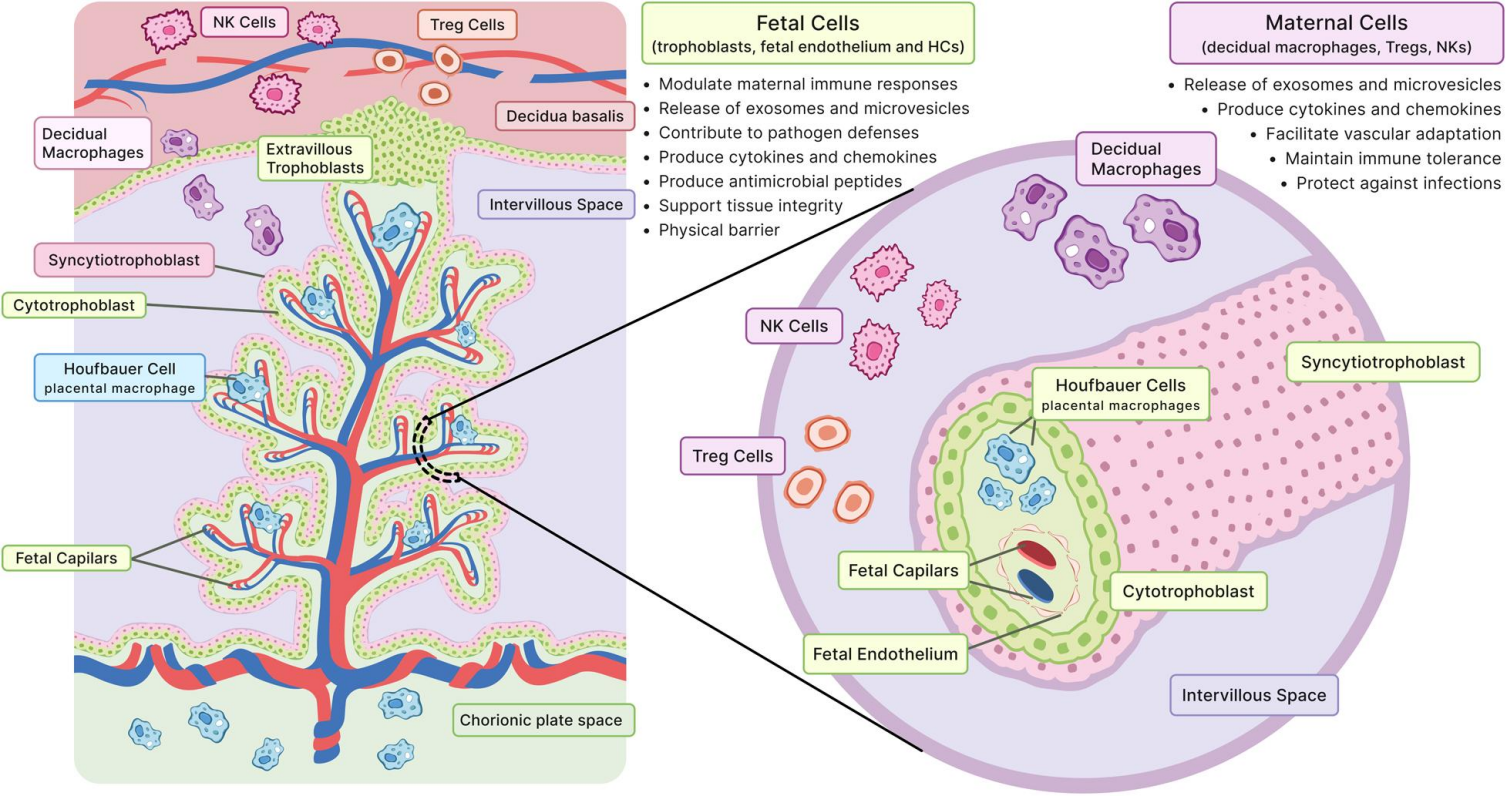
Maternal–fetal immune crosstalk at the placental villus. **Left:** Decidua and intervillous space, showing maternal immune cells (decidual macrophages, Tregs, and uterine NKs) interacting with extravillous trophoblasts and the villous tree. **Right:** Magnified cross-section of the villus. The structure includes the syncytiotrophoblast (outer layer), cytotrophoblast (inner layer), and villous stroma. The stroma contains Hofbauer cells (fetal macrophages) and fetal capillaries lined by endothelium. These components are bathed in maternal blood within the intervillous space. NK, natural killer cell; Treg, regulatory T cell.

Placental immunity involves a coordinated interaction between maternal and fetal immune cells to maintain immune tolerance at the maternal–fetal interface, protect against infections, and ensure appropriate nutrient and oxygen transfer to the fetus (69). Maternal cells such as decidual macrophages, T regulatory cells (Tregs), and natural killer (NK) cells promote tolerance, protect against infections, and facilitate vascular adaptation (70, 71). Fetal cells, including trophoblasts and Hofbauer cells (placental macrophages), modulate maternal immune responses, support tissue integrity, and contribute to pathogen defenses (72). Trophoblasts also create a physical barrier and produce antimicrobial peptides to prevent pathogen transmission (73, 74). Both maternal and fetal immune cells in the placenta produce cytokines and chemokines to communicate, and they release exosomes and extracellular vesicles to further regulate immune responses (Figure 4) (75).

During normal pregnancy, placental immunity is characterized by a finely tuned balance between tolerance and defense. Decidual NK cells promote vascular remodeling while exhibiting limited cytotoxic activity, regulatory T cells suppress excessive maternal immune activation, and decidual macrophages adopt predominantly tissue-remodeling and anti-inflammatory phenotypes that support trophoblast invasion and spiral artery transformation. Trophoblasts further contribute by restricting expression of classical HLA molecules and secreting immunomodulatory factors that dampen maternal effector responses. Together, these mechanisms establish a controlled immune environment that permits fetal tolerance while preserving antimicrobial capacity (76–78).

Disruption of this coordinated regulatory network shifts the placental immune environment from controlled adaptation to maladaptive inflammation, a condition increasingly associated with adverse pregnancy outcomes (13, 79). Inflammatory activation may be triggered by infectious exposures, including viral (HIV, CMV, Zika virus) (80, 81), bacterial (chorioamnionitis, Group B Streptococcus) (82), and parasitic infections (*Plasmodium* spp, *Toxoplasma gondii*) (83, 84), as well as noninfectious etiologies such as autoimmune activation, environmental toxicants, poor nutrition, and maternal stress (85). These diverse exposures converge on stress-responsive and innate immune pathways, including *NF-κB* signaling, leading to cytokine production and immune cell recruitment (79, 86, 87). The resulting inflammatory response disrupts trophoblast invasion and spiral artery remodeling, impairs endothelial function, and compromises nutrient and oxygen exchange (63, 88). In parallel, interactions with oxidative stress and anti-angiogenic pathways generate feed-forward inflammatory cycles that exacerbate vascular malperfusion and syncytiotrophoblast stress (89), contributing to placenta-mediated complications such as preeclampsia, preterm birth, fetal growth restriction and stillbirth (13, 90).

Importantly, placental inflammation does not play a single, linear role in pregnancy complications. It may function as a biological imprint of cumulative exposures, a mediator of placental dysfunction, or part of amplifying inflammatory cycles. For example, maternal psychosocial stress and cumulative allostatic load are associated with inflammatory activation during pregnancy even in the absence of overt infection (91), supporting their role as markers of physiological embedding. At the same time, mechanistic studies demonstrate that inflammatory activation directly contributes to vascular maladaptation and trophoblast dysfunction (72, 92). The relative contribution of these roles likely varies by gestational timing, exposure type, and maternal cardiometabolic context. This layered model helps reconcile observational associations with mechanistic data and underscores the importance of temporally resolved and causally informed placental research.

Studies report a higher prevalence of placental inflammatory lesions among African American women compared with European American women, even after adjustment for measured social determinants of health (86, 93, 94). These inflammatory patterns parallel increased rates of pregnancy-associated hypertension, preterm birth, and small-for-gestational-age birth (95). Within this framework, a higher inflammatory burden may reflect both disproportionate upstream exposures and differential amplification of inflammatory pathways that contribute to placenta-mediated complications. Clarifying how placental inflammation operates remains essential for disentangling biological susceptibility from socially patterned exposure and for designing equity-focused preventive strategies.

## 6 The impact of race, self-identified ethnicity, and ancestry in pregnancy research

The continued reliance on racial categories in human genetics undermines efforts to address health inequities among African American pregnant women (43, 96–98). This approach is problematic because race is not a valid biological concept; it fails to capture the extensive genetic diversity, social-environmental exposure, and continuous variation within and between populations (97, 99). The use of race as a marker in scientific research conflates environmental and socioeconomic variables with biological variables (96, 100). This conflation obscures the distinct genetic and nongenetic contributions to health outcomes and can lead to misleading conclusions (101). Furthermore, the continued use of race can perpetuate outdated and harmful scientific bias (102). Instead, focusing on genetic ancestry and self-identified ethnicity offers a more precise, ethical, and detailed approach to understanding human genetic variation alongside historical and socioenvironmental influences on epigenetics (100).

Genetic ancestry is the proportion of an individual's genome inherited from different ancestral lineages. The Human Genome Project and subsequent genetic research have demonstrated that humans share a high degree of genetic similarity, with 99.9% of our DNA being identical (103). The minor genetic variations that do exist do not align neatly with racial categories. Instead, they reflect a continuum of genetic diversity shaped by historical migrations and interbreeding (96). Ancestry estimation approaches leverage specific genetic markers, known as ancestry-informative markers, which vary in frequency across populations and can reveal the complex historical migrations and admixture events that shaped contemporary human populations (104).

Nevertheless, an individual's self-identified ethnicity remains relevant in any comprehensive analysis of health inequities (96). Although self-identification does not correspond neatly to genetic ancestry, it often captures unique life experiences, such as cultural practices, historical trauma, exposure to discrimination, and chronic stress, that can influence epigenetic processes and, ultimately, health outcomes (27). For example, African Americans whose families were brought to the U.S. through slavery may have endured different intergenerational stressors than individuals who more recently immigrated voluntarily. Alternatively, a person who is not genetically of African ancestry but who lives within an African diaspora community in the U.S. might share many of the same lived experiences that can shape epigenetic patterns. Although using race as a proxy for biological variation is problematic, ignoring self-identified ethnicity and its potential epigenetic impact is also unwise (96).

In practice, self-identified ethnicity should be incorporated as a structured component of health history rather than treated solely as a demographic variable. When interpreted within a structural and sociohistorical context, it can help contextualize cumulative exposures, including discrimination, neighborhood-level environmental risk, access to health care, nutritional patterns, and intergenerational stress. These exposures influence placental function and maternal-fetal outcomes independently of genetic ancestry (105, 106).

Hence, disentangling genetic ancestry from the socioenvironmental factors associated with ethnicity is crucial for understanding pregnancy outcomes in a nuanced way. By teasing apart inherited genetic variation from lived experiences such as discrimination, cultural practices, and historical trauma, scientists can design more rigorous studies, interpret data more accurately, and develop targeted interventions that address both genetic predispositions and the social determinants of health. Together, this integrated approach avoids oversimplification and provides deeper insight into how the intersection of biological inheritance and lived social experience shapes placental function and pregnancy outcomes.

## 7 Genetic variation in placenta-mediated complications: state of the science in African-ancestry populations

Genetic architecture varies across ancestries due to differences in allele frequencies and linkage disequilibrium structure, directly influencing locus discovery, fine-mapping resolution, and the portability of risk estimates (107). Additionally, findings established in one ancestry often do not transfer cleanly to another. Without adequate African-ancestry representation, we risk missing causal variants, mislocalizing signals, and building tools that may underperform in populations bearing disproportionate disease burden (43). In contrast, adding diverse cohorts, especially African ancestry populations, boosts power, sharpens credible sets via linkage disequilibrium contrasts, and reveals loci missed in homogeneous designs (108, 109).

**Figure 5:**
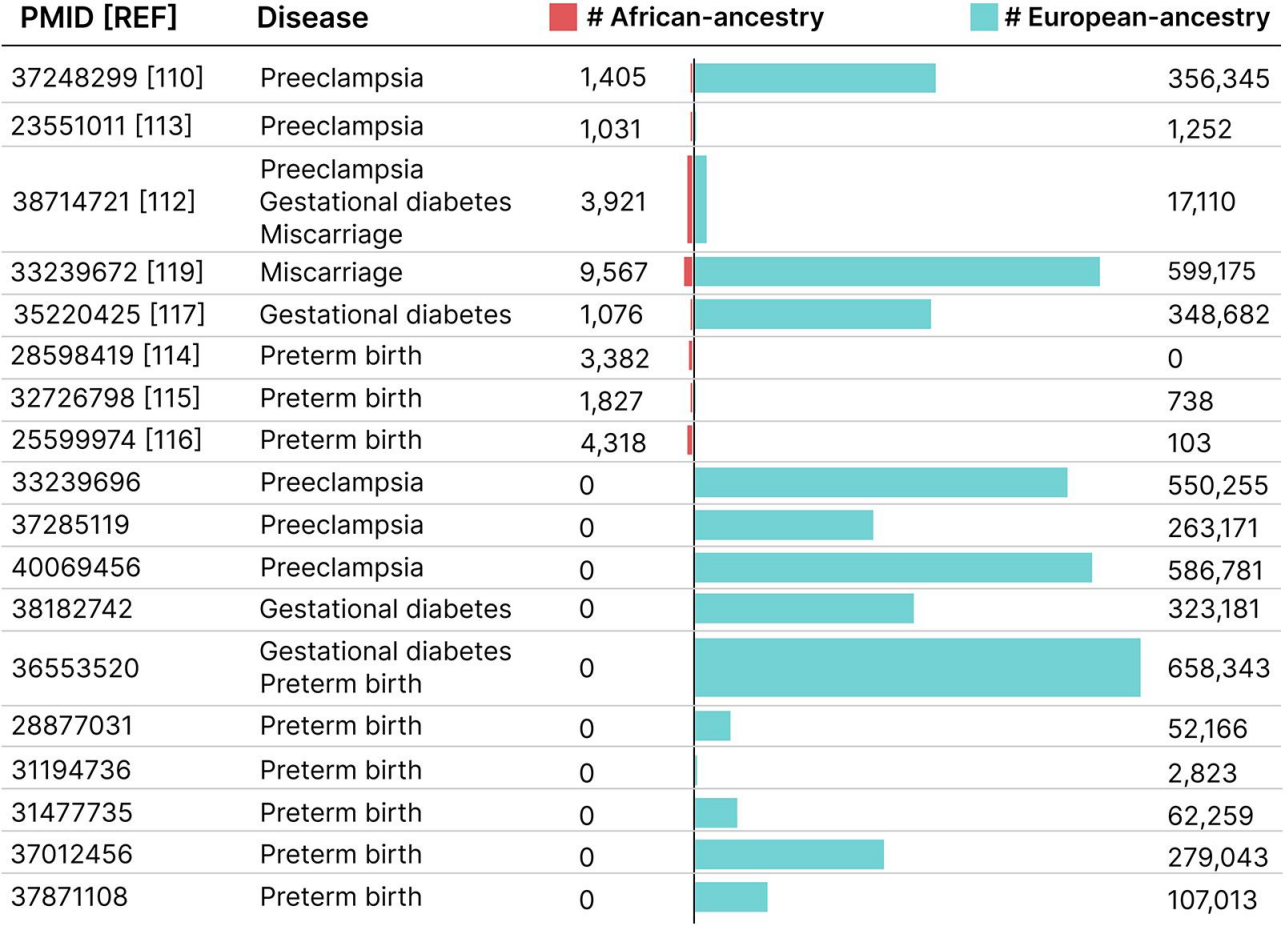
African and European ancestry composition of GWAS across pregnancy diseases (preeclampsia, gestational diabetes, miscarriage, preterm birth). Horizontal bars show the sample sizes for African- and European-ancestry samples per study. Counts are from the GWAS Catalog except for PMID 37248299 (110), where we use manuscript-reported, disease-specific totals. GWAS Catalog metadata may conflate discovery and replication, and maternal and fetal strata.

To assess underrepresentation in placenta-mediated complications, we queried the GWAS Catalog for preeclampsia, preterm birth, gestational diabetes, and miscarriage, retaining only studies including African- and/or European-ancestry participants (Figure 5). Supplementary Table S1 summarizes lead loci, effect sizes, *p*-values, and ancestry composition for the African-inclusive studies identified. A clear structural imbalance emerges. In large multi-biobank analyses of hypertensive disorders of pregnancy (110), African-ancestry representation was typically less than 1%–3% in discovery cohorts, while European-only studies frequently exceeded 300,000–600,000 participants. Similarly, trans-ancestry gestational diabetes meta-analyses (111) included only approximately 1%–2% African ancestry. Several analyses labeled “multi-ancestry” were overwhelmingly European-dominant in discovery phases, limiting power to detect ancestry-enriched effects.

For gestational diabetes mellitus (GDM), multi-ancestry GWAS meta-analysis with meta-regression (MR-MEGA) (111) identified established loci including MTNR1B, TCF7L2, CDKAL1, CDKN2A–CDKN2B, and HKDC1, with African-ancestry representation of approximately 1.7%. Although effect directions were broadly consistent across strata, limited African-ancestry sample size constrained heterogeneity testing and ancestry-specific fine-mapping. The nuMoM2b study (112), which implemented ancestry-stratified GWAS followed by meta-analysis (with approximately 16%–19% African ancestry, depending on phenotype), represents methodological progress. Genome-wide significant loci were identified for GDM (e.g., ZBTB20, RPL7P20, GUCY1A2) and gestational length. However, locus-level contributions varied by ancestry, and preeclampsia yielded no genome-wide significant loci despite improved representation, underscoring persistent power limitations.

Large maternal GWAS meta-analyses of preeclampsia and gestational hypertension (109) illustrate the distinction between “multi-ancestry” labeling and balanced representation. Discovery cohorts were overwhelmingly European-dominant, with <1% African ancestry. Identified loci, including FLT1, FGF5, MECOM, SH2B3, and cardiometabolic-related regions, largely overlap established blood pressure and vascular pathways, raising concerns about the cross-ancestry portability of findings derived from European-dominant discovery. In contrast, the nuMoM2b study (112), despite more balanced representation, did not identify genome-wide significant loci for preeclampsia, highlighting the need for substantially larger harmonized cohorts for robust mapping of this phenotype across ancestries. Similarly, an early multi-ethnic GWAS of preeclampsia in the HAPO cohort (113) included Afro-Caribbean, European, and Hispanic mothers but contained only 21 cases in the African-ancestry subset. Although suggestive associations were reported (e.g., variants near FGF14 and MYCBP2), no loci reached genome-wide significance, reflecting the limited statistical power of early African-inclusive studies.

Preterm birth provides one of the few examples of African-ancestry-centered discovery. A genome-wide association study conducted in African American women from the Boston Birth Cohort (114) identified loci associated with spontaneous preterm birth and gestational length, including signals at 8p12 and in the HGF region. A subsequent gene–environment interaction GWAS in the same cohort (115) identified a genome-wide significant interaction at PTPRD modified by maternal stress exposure. Prospective case-control GWAS from the NICHD Genomic and Proteomic Network for Preterm Birth Research (GPN-PBR) (116), which included ∼23% African ancestry, identified loci for early spontaneous preterm birth (<34 weeks), including DCP1A, RNASET2, and an MHC-region signal. These studies demonstrate that meaningful locus discovery becomes possible when African-ancestry representation exceeds marginal inclusion thresholds, although modest sample sizes constrain replication stability and precision.

In miscarriage, large European-only meta-analyses (117) identified loci such as NAV2/E2F8 and intergenic signals on chromosomes 9, 21, and 2. African-ancestry representation was absent or unquantified in most discovery analyses, thereby limiting the resolution of trans-ethnic fine-mapping. Trans-ethnic meta-regression frameworks (MR-MEGA) were implemented in some analyses (117), but interpretation remained constrained by limited African sample size. In nuMoM2b (112), genome-wide significant loci for pregnancy loss (RGMA, TRMU) were identified in European plus African meta-analysis, although ancestry-specific contributions varied, and some strata were excluded due to low case counts.

Collectively, current GWASs of placenta-mediated complications reveal a field still constrained by ancestry imbalance. While loci for gestational diabetes and hypertensive disorders of pregnancy have been identified in largely European-dominant analyses, African-centered studies demonstrate that additional signals can emerge when representation improves. Expanding harmonized cohorts with substantial African-ancestry participation will therefore be essential to clarify shared vs. ancestry-enriched genetic architecture and to improve the generalizability of genetic risk estimates across populations.

## 8 Epigenetic mechanisms modulating placental health

Epigenetics refers to heritable, stable molecular modifications that regulate gene expression without altering the DNA sequence (118). In the placenta, these mechanisms enable cells to integrate environmental signals and maintain memory of prior exposures over time (119). Major epigenetic mechanisms include DNA methylation, histone modifications, and noncoding RNAs (120). Together, they regulate fundamental placental programs, including transcription, genomic imprinting, X-chromosome inactivation, retrotransposon regulation, and genomic stability (121). Genomic imprinting is particularly prominent in the placenta and establishes parent-of-origin–specific expression patterns at loci regulating growth, nutrient allocation, and endocrine function. Many imprinted genes exhibit preferential paternal expression, underscoring that placental epigenetic regulation reflects contributions from both parental genomes (122).

**Figure 6:**
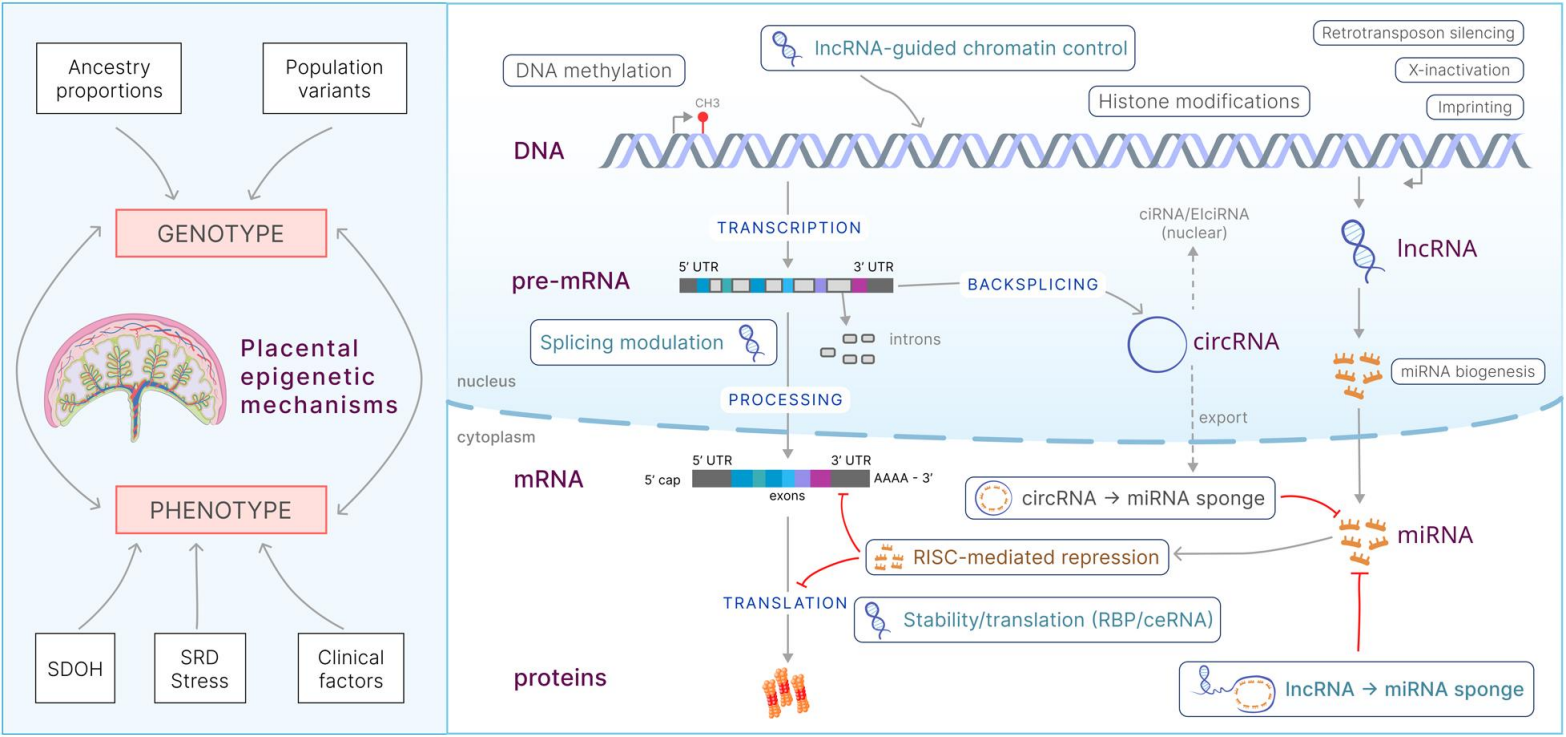
Placental epigenetic mechanisms. **Left**: Genotype (ancestry proportions and population variants) and phenotype (social determinants of health, structural racism/discrimination, stress, and clinical factors) converge on placental epigenetic mechanisms. **Right**: Schematic of placental epigenetic mechanisms. SDOH, social determinants of health; SRD, structural racism and discrimination; RBPs, RNA binding proteins; lncRNA, long noncoding RNA; circRNA, circular RNA; miRNA, microRNA; RISC, RNA-induced silencing complex; ceRNA, competing endogenous RNA.

The placenta is the central hub of epigenetic modulation during prenatal development, integrating paternal and maternal genetic and environmental inputs to shape fetal growth and long-term health outcomes (Figure 6) (123, 124). Although epigenetic regulation is essential for placental development, the specific mechanisms by which these modifications dynamically respond to environmental signals remain incompletely understood and an area of active research. Furthermore, placental epigenetic modifications may influence neurodevelopment by integrating early-life experiences into developing brain circuits, potentially leading to long-term changes in neurobiology and behavior (125).

DNA methylation, catalyzed by DNA methyltransferases, involves the addition of a methyl group to cytosine residues, typically within CpG dinucleotides. When present in promoter-associated CpG islands, methylation represses transcription by inhibiting transcription factor binding or by recruiting methyl-binding proteins that promote chromatin compaction (126). In the placenta, DNA methylation regulates trophoblast differentiation, placental invasion, nutrient transport and genomic imprinting (127–129). It also mediates X-chromosome inactivation in female placentae (130). Environmental exposures, including toxins and maternal stress, can alter DNA methylation patterns, contributing to adverse pregnancy outcomes such as preeclampsia and fetal growth restriction (131).

Histone modifications regulate chromatin architecture and gene accessibility. Posttranslational modifications, including acetylation, methylation, phosphorylation, and ubiquitination, alter nucleosome structure and recruit regulatory complexes (132). During placental development, histone modifications control genes that guide the differentiation and function of trophoblast cells. For example, the polycomb repressive complex 2 (PRC2) mediates the trimethylation of histone H3 at lysine 27 (H3K27me3), maintaining the repression of developmental genes in the placenta (133). Appropriate histone acetylation patterns are also vital for supporting trophoblast differentiation and placenta development (134).

Noncoding RNAs (ncRNAs), regulate gene expression at transcriptional and posttranscriptional levels and are particularly important in placental development. MicroRNAs (miRNAs), approximately 22 nucleotides in length, bind target mRNAs to induce degradation or translational repression (135). One notable example is miR-675, derived from the H19 lncRNA, which regulates placental growth by targeting the insulin-like growth factor 1 receptor (*IGF1R*), thereby balancing growth-promoting and growth-inhibitory signals (136). Canonically, miRNAs assemble with Argonaute (AGO2) in the RNA-induced silencing complex (RISC) and bind complementary sites—most frequently in 3′UTRs—leading to deadenylation, decapping, and mRNA decay. In some contexts, they primarily block translation initiation/elongation without decay. Placenta-enriched miRNA clusters (e.g., C19MC and C14MC) exemplify this layer of control (137, 138).

Long noncoding RNAs (lncRNAs), which are longer than 200 nucleotides, regulate gene expression at multiple levels, including chromatin modification, transcription, and posttranscriptional processing (139). For example, the H19 lncRNA, in addition to being the precursor of miR-675, modulates neighboring imprinted genes such as *IGF2*. This regulation ensures the balance between growth-promoting and growth-inhibiting signals in the placenta (140). Circular RNAs (circRNAs), generated by backsplicing, frequently act as competing endogenous RNAs by sponging specific miRNAs, thereby derepressing the corresponding mRNAs. CDR1as/CiRS-7 is a circular RNA that tightly binds to miR-7 and modulates its activity. It contains dozens of miR-7 seed sites and is detected in approximately 59% of placental RNA-seq datasets (34).

Additional epigenetic mechanisms essential to the placenta include chromatin remodeling and DNA hydroxymethylation. Chromatin remodeling complexes such as SWI/SNF and NuRD facilitate transcriptional accessibility during processes such as trophoblast invasion (141). DNA hydroxymethylation, catalyzed by TET enzymes, further refines gene regulation during placental differentiation and nutrient transfer (142). Importantly, epigenetic layers in the placenta also operate in coordinated networks. For example, lncRNAs such as *XIST* recruit histone-modifying enzymes to specific genomic loci, influencing chromatin states and gene expression (143). Similarly, DNA methylation can recruit methyl-CpG-binding domain (MBD) proteins that interact with histone deacetylases to reinforce transcriptional repression. This integrated regulation ensures that gene expression is tightly regulated to support the intricate process of placental development and environmental adaptation (144).

## 9 Impact of environmental and socioeconomic factors on placental epigenetics

**Figure 7:**
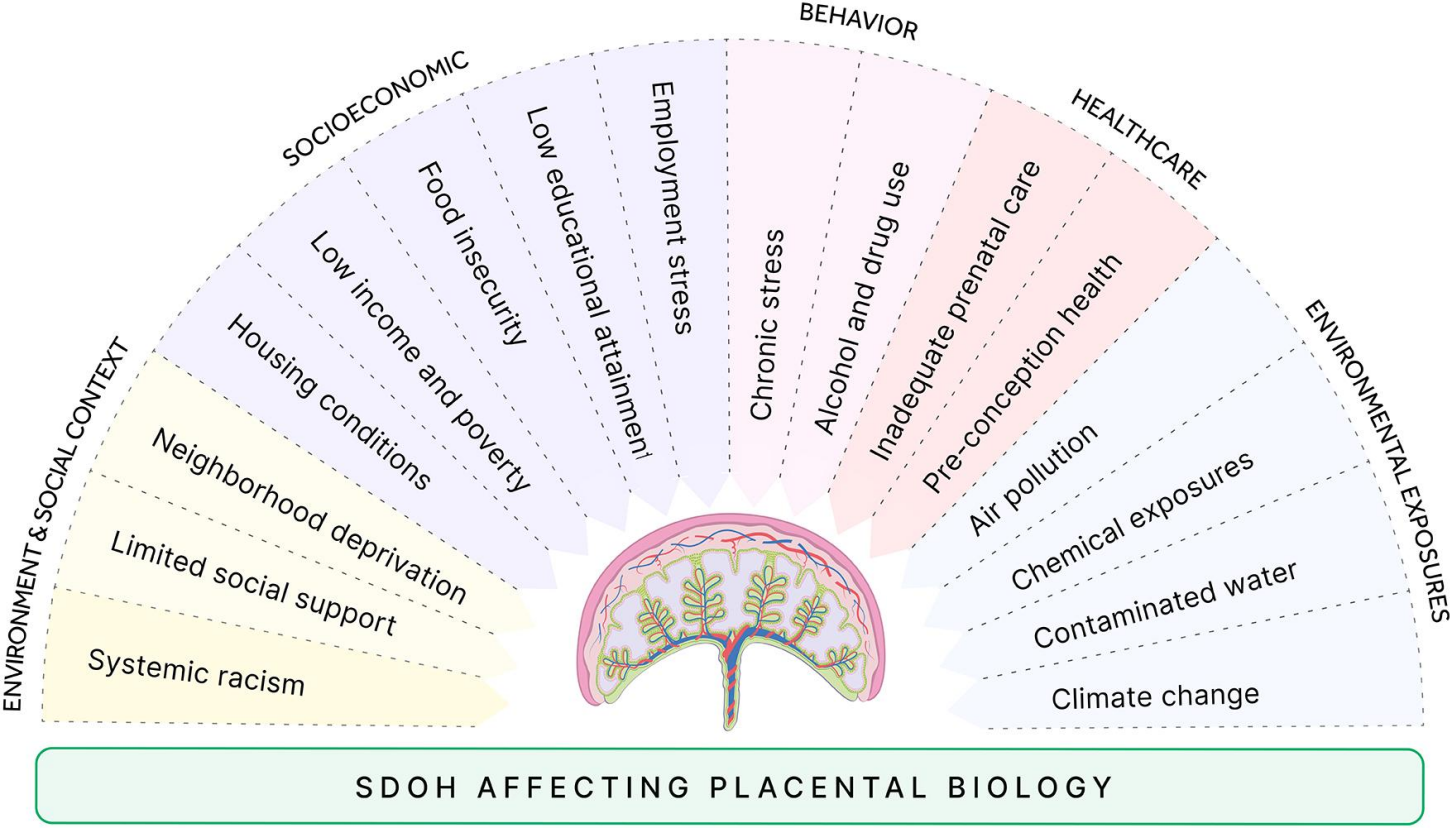
Social determinants of health (SDOH) affect placental biology. Summary of five upstream domains (environment and social context, socioeconomic conditions, behaviors, healthcare access/quality, and environmental exposures) with representative examples. These factors can act alone and interact to modify placental pathways and, ultimately, pregnancy outcomes. (Examples are illustrative, not exhaustive.).

Placental epigenetic programs are at the core of the developmental origins of health and disease (DOHaD) model, translating social and environmental exposures into fetal development and long-term risk, which are burdens that fall disproportionately on African American women and contribute to adverse pregnancy outcomes (145). These factors encompass a broad range of variables (Figure 7). Socioeconomic variables include low income and poverty, food insecurity and malnutrition, low educational attainment, employment and occupational stress, and inadequate healthcare and prenatal care (146). The environmental variables include poor housing conditions, neighborhood deprivation, limited social support, systemic racism, and discrimination (147, 148). Chronic stress, ethanol and drug use, and overall health during the preconception phase can also have profound effects, influencing both maternal and fetal health (149, 150). Additionally, air pollution, chemical exposure, contaminated water, and climate change further exacerbate the risks (151, 152).

While DOHaD research has historically emphasized maternal exposures, emerging evidence suggests that paternal preconception environment may also shape early developmental trajectories through epigenetic information carried in sperm, including DNA methylation patterns, retained histones, and small noncoding RNAs (38). Although extensive epigenetic reprogramming occurs after fertilization and the persistence of specific paternal marks in humans remains under investigation, these mechanisms represent a potential upstream pathway through which paternal exposures may intersect with placental epigenetic programming. For example, human studies have shown that paternal obesity is associated with altered sperm DNA methylation at imprinted loci implicated in growth regulation, including IGF2, and that substantial weight loss can remodel the sperm methylome, supporting the plausibility of environmentally responsive paternal epigenetic programming prior to conception (153). Understanding how these variables influence epigenetic modulation of gene expression in placental cells provides insights to improve maternal and fetal health and address health inequities.

Natural experiments and cohorts show how specific maternal exposures map to placental pathways. The seminal study by Tobi and collaborators on the Dutch Hunger Winter demonstrated that early-gestation famine leaves persistent differentially methylated regions (DMRs) in the placenta, affecting growth and metabolic pathways. Six placental DMRs were subjected to technical and biological validation and mapped to the genes *SMAD7*, *CDH23*, *INSR*, *RFTN1*, *CPT1A*, and *KLF13*, which support durable embedding of early adversity (154).

Many studies have advanced our understanding of how epigenetic mechanisms impact placental health and pregnancy outcomes. In a case–control study of term placentas, maternal anxiety/depression and stressful life events were associated with methylation and transcriptomic shifts in vascular/ECM and ER-protein-processing programs, with downregulation of ribosome/EMT/DNA-repair pathways, signals consistent with impaired placental remodeling and resilience (106, 155). A study involving 903 mother‒child pairs from the Boston Birth Cohort, an urban, low-income minority cohort, examined how maternal prepregnancy BMI influences DNA methylation in newborns and its subsequent impact on childhood obesity. The study revealed that higher prepregnancy BMI is associated with newborn methylation signatures at loci linked to energy balance and metabolic syndrome. In addition, these signatures partly mediate childhood obesity risk (156).

In a cohort of 301 low-risk pregnancies, increased social support was associated with methylation/expression shifts in genes governing neurodevelopment and metabolism (notably *VGF* and *ILVBL*), and functional analysis implicated fetal growth, coagulation, energy metabolism, and neurodevelopment (157). In 1,029 mother–child pairs, prenatal stress and maternal childhood trauma were associated with increased placental *ADGRG6* and reduced *RAB11FIP3*/*SMYD5* expression. Pathway analyses revealed upregulated ER protein-processing/secretory and ubiquitin–proteolysis programs, alongside downregulated ribosome, EMT, DNA repair, MYC-target, and amino-acid metabolism pathways, indicating disrupted placental remodelling (106). However, few studies have examined whether these stress-associated epigenetic changes vary by genetic ancestry or interact with ancestry-linked regulatory architecture.

## 10 Ancestry-associated variation in placental epigenomes

Variation in placental epigenomes across ancestral backgrounds may contribute to differences in pregnancy risk. Analyses disentangling ancestry from altitude (Andeans vs. Europeans) indicate that ancestry-associated differences are concentrated in inflammatory and angiogenic pathways, whereas altitude-related changes converge on AP-1/hypoxia-responsive networks (158). In addition, a high-altitude-tuned variant in *DYSF* modulates placental methylation, and its expression shows balancing selection at high altitude, consistent with its roles in syncytiotrophoblast turnover/repair and illustrating how ancestry and the environment combine in placental adaptation (159).

Emerging evidence suggests that genetic ancestry and socially patterned exposures jointly shape the placental epigenome. DNA methylation is partly regulated by methylation quantitative trait loci (meQTLs), in which genetic variants influence local methylation levels and downstream gene expression. Because allele frequencies differ across ancestral backgrounds, these regulatory variants can contribute to systematic differences in methylation profiles across populations (106, 160). In admixed populations, local ancestry at specific genomic regions may further influence regulatory activity (160).

Epigenome-wide studies provide empirical support for these mechanisms. Analyses of DNA methylation across ancestry-diverse cohorts have shown that a substantial fraction of differentially methylated CpG sites between populations can be explained by underlying genetic variation through meQTLs, demonstrating that ancestry-associated genetic variation contributes to epigenomic regulation (160). Similarly, regulatory mapping studies in human placental tissue have identified genetic effects on molecular phenotypes, including DNA methylation and gene expression, at loci involved in immune signaling, angiogenesis, and metabolic regulation—processes central to placental vascular remodeling and nutrient exchange (161). Additional placental epigenetic studies examining pregnancy outcomes have linked differential methylation patterns to pathways associated with fetal growth and cardiometabolic risk, highlighting how epigenomic variation may contribute to population-level differences in pregnancy outcomes (162).

At the same time, environmental exposures also shape placental methylation patterns. Maternal stress, metabolic status, and environmental adversity have been associated with measurable epigenetic changes in placental tissue (163–165), reflecting the biological embedding of socially patterned exposures. Few studies currently integrate genetic ancestry, regulatory genetic variation, detailed exposure measurements, and functional placental outcomes within a single analytical framework. Disentangling inherited regulatory variation from exposure-driven epigenetic remodeling therefore remains an important frontier for understanding population-level differences in placental function and pregnancy risk.

One approach to capturing the cumulative effects of genetic background and environmental exposures on the epigenome is to use epigenetic aging metrics. Epigenetic age, derived from DNA methylation profiles, estimates biological aging rather than the chronological age. It captures the cumulative effects of lifestyle, environment, and genetic background and has been linked to future disease risk and mortality (166). Recent work has reported faster epigenetic aging among African American participants, which was attributable in part to greater exposure to stressful and traumatic life events, even after adjustment for lifestyle and demographics, which is consistent with the biological embedding of stress (167). These findings suggest that observed differences in epigenetic aging reflect cumulative exposure to structurally patterned stressors rather than inherent biological distinctions. Because genetic ancestry shapes epigenetic and transcriptomic variation, studies of epigenetic aging should incorporate genetic ancestry alongside self-identified ethnicity to disentangle inherited from socioenvironmental contributions across diverse populations (168, 169).

Similarly, placental epigenetic age at birth is a readout of prenatal conditions (10). In the NICHD Fetal Growth Study, a 62-CpG placental clock revealed that placental epigenetic age acceleration (PAA) varies with maternal cardiometabolic status and genetic ancestry (170). In male placentas, greater gestational weight gain, higher first-/third-trimester blood pressure, and prepregnancy obesity are associated with lower PAA. Among Hispanics, greater Native American ancestry is associated with higher PAA, whereas greater African ancestry is associated with lower PAA. Among Asians, greater East Asian ancestry is associated with lower PAA (170). These findings imply that the maternal metabolic milieu and inherited background modulate the tempo of placental aging. Integrative models incorporating local ancestry, regulatory genetic architecture, placental epigenomic remodeling, transcriptional programs, temporally resolved social and environmental exposures, and clinical phenotypes are needed. Such multimodal approaches can clarify causal pathways, refine early risk stratification, and support equitable precision medicine during pregnancy.

## 11 Pregnancy data collection and artificial intelligence modelling

Machine learning and AI methods hold significant potential to improve maternal-fetal research because pregnancy is a time-structured biological process with well-defined physiological stages and clinical decision points (171). These approaches can integrate ancestry-informed genomics, placental multi-omics, EHRs, imaging, and SDoH to support earlier, more precise, and more equitable risk prediction and intervention (172). However, realizing this promise requires confronting risks that mirror the structural inequities documented throughout this review. AI is not a shortcut but an amplifier: when trained on sparse or skewed data it can widen existing gaps, whereas models built on representative, well-governed, placenta-aware datasets can translate multidimensional biological and clinical information into earlier detection, more appropriate surveillance, and timely intervention for those at highest risk.

Hypertensive disorders of pregnancy illustrate the translational potential of these approaches. In the largest multi-ancestry GWAS of preeclampsia and gestational hypertension to date, maternal loci including *FLT1*, *FGF5*, and *SH2B3* were identified, and polygenic risk scores derived from these discoveries modestly improved early risk classification when combined with clinical predictors such as body mass index and blood pressure (110). In that same study, however, polygenic risk scores performance was attenuated in individuals of African ancestry, reflecting reduced portability of polygenic prediction across ancestral groups (115). This observation is consistent with broader evidence showing that polygenic scores for cardiometabolic traits, which share genetic architecture with hypertensive disorders of pregnancy, can exhibit two- to five-fold reductions in predictive accuracy in African-ancestry populations when derived primarily from European-ancestry datasets (173, 174). In clinical settings, such attenuation may translate into systematic risk under- or overestimation in populations already experiencing disproportionate maternal morbidity.

EHR-based machine learning models further illustrate both opportunity and constraint in obstetric prediction. Models trained on longitudinal prenatal EHR data have demonstrated that routinely collected demographic, diagnostic, and laboratory variables can predict preterm birth months before delivery with strong internal validation performance (175). Similarly, early prediction of preeclampsia using structured EHR variables has shown favorable discriminative performance in single-health-system cohorts (176). However, these models often rely heavily on institution-specific coding practices and the completeness of gestational data capture, and external validation across independent healthcare systems remains limited (176). More broadly, clinical prediction models frequently experience performance degradation when transported across institutions due to dataset shift, differences in documentation structure, and differences in population composition (177, 178). Together, these observations indicate that in pregnancy research, model performance is shaped as much by data architecture and phenotype harmonization as by algorithm selection.

Equitable implementation of AI in maternal–fetal research aligns with established methodological guidance from clinical prediction and machine learning communities. Reporting and development standards such as TRIPOD and its AI extension (TRIPOD-AI), CONSORT-AI, and SPIRIT-AI emphasize transparent reporting, prespecified outcomes, and rigorous external validation prior to clinical deployment (179–181). These frameworks recommend explicit documentation of data provenance, eligibility criteria, missingness patterns, and performance metrics beyond discrimination, including calibration and subgroup analyses.

In pregnancy research, best practice therefore includes harmonized gestational phenotype definitions, ancestry-stratified training and validation, and reporting of model performance across clinically relevant subgroups rather than pooled metrics alone (179–181). Broader clinical AI guidance further emphasizes evaluation under dataset shift and ongoing monitoring after deployment, including drift detection and recalibration as population characteristics or screening protocols evolve (175, 176). Regulatory and ethical bodies, including the World Health Organization and the U.S. Food and Drug Administration, similarly emphasize that clinical AI tools must demonstrate transparency, fairness, and post-market performance surveillance before and after integration into healthcare pathways (182, 183). Together, these recommendations provide a structured framework for the responsible implementation of AI in maternal–placental medicine. Ultimately, predictive accuracy alone is insufficient. Consistent with consensus standards in clinical AI evaluation, success should be judged by whether deployment improves timely intervention while reducing—rather than exacerbating—disparities in maternal and fetal outcomes.

An equity-by-design approach is therefore essential for AI in gestational research. Research cohorts must intentionally reflect the populations most affected by adverse pregnancy outcomes, including appropriate representation of historically underrepresented populations. Such approaches should integrate ancestry-informed genomics alongside SDoH (173), and incorporate standardized protocols for placenta procurement, phenotyping, and gestational metadata collection (30). Transparent governance of data use, community engagement in research design, and the use of privacy-preserving analytical frameworks are equally critical to maintaining trust and accountability in maternal-fetal AI research (179–181).

Before clinical deployment, predictive models should demonstrate robust external and prospective validation with appropriate subgroup calibration (179–181). Following deployment, systems require continuous monitoring for performance drift, scheduled recalibration, and prespecified fairness audits to ensure that predictive accuracy and equity are maintained across evolving patient populations (177, 178). Rigorous adherence to these methodological and ethical standards is necessary to ensure that computational innovations ultimately reduce, rather than reinforce, existing inequities in maternal and fetal health outcomes.

## 12 Discussion

Maternal-fetal health inequities arise from several controversies and methodological tensions that continue to shape maternal–fetal research and healthcare. A central debate concerns the persistent use of racial categories in biomedical research. Because race is not a biological variable, reliance on race as a proxy for biology can obscure inference and conflate inherited variation with socially patterned exposures. Furthermore, across domains—genomics, epigenomics, and machine learning—the same structural gaps repeatedly constrain clinical translation. Placental cohorts remain small, limited to late gestation, and often lack in-depth phenotyping of exposures and outcomes. Underrepresentation of African-ancestry populations persists across genomic cohorts, reducing power for discovery and fine-mapping and weakening the portability of risk estimates precisely where the burden is highest. Methodological inconsistencies in placental sampling, analysis, and exposure measurement further limit cross-study synthesis. Finally, computational models developed on underrepresented data are at risk of amplifying existing inequities rather than mitigating them.

Three priorities follow from this review. First, the field must close the placenta data gap with rigorous, standardized collection and deep phenotyping across gestation, paired with genome references and analytic approaches that work well in diverse populations, including the use of pangenome reference assemblies, trans-ancestry genome-wide association studies, ancestry-aware fine-mapping methods that leverage differences in linkage disequilibrium, and local ancestry–informed analyses. Second, studies must include genetic ancestry analysis alongside self-identified ethnicity and carefully measured social and environmental exposures to capture the lived conditions that drive biological embedding. Third, AI and precision medicine will only improve outcomes if models are trained on representative data with intentional oversampling of high-risk groups, validated prospectively, audited for subgroup performance, and implemented with accountability and in partnership with communities. Together, these priorities underscore the need to reframe placental research within a broader biological and clinical context.

Ultimately, the placenta should not be viewed merely as a transient organ of pregnancy, nor should pregnancy complications be considered confined to obstetric care alone. Conditions such as preeclampsia, fetal growth restriction, and preterm birth have lasting consequences for maternal cardiovascular health, offspring metabolic risk, and intergenerational disease transmission. Placental dysfunction, therefore, represents not only a pregnancy-specific pathology but a central human health challenge, with implications that extend beyond gestation and across the life course. Despite its central biological role in shaping lifelong health trajectories, the placenta remains comparatively under-characterized within genomic frameworks and translational research infrastructures. Addressing this gap is both scientifically urgent and feasible. By centering placental biology within inclusive, ancestry-informed, and methodologically rigorous research designs, maternal–fetal medicine can advance toward predictive, preventive, and equity-oriented strategies that reflect the full biological and social complexity of human reproduction.

## Supplementary material

SUPPLEMENTARY TABLE S1 Genome-wide association studies of placenta-mediated pregnancy complications that include participants of African ancestry. This table summarizes genome-wide association studies (GWAS) investigating placenta-mediated pregnancy complications—including preeclampsia, preterm birth, gestational diabetes, and related maternal traits—that include participants of African ancestry. For each study, we report the lead locus, lead variant, effect size, statistical significance, ancestry representation, and analytical strategy used to account for population structure. Despite increasing recognition of the importance of diverse genomic datasets, African-ancestry representation in these studies remains limited relative to European-ancestry cohorts, highlighting a persistent imbalance in genomic research and the need for expanded inclusion to improve locus discovery, fine-mapping resolution, and the generalizability of genetic findings. AFR, African ancestry; GWAS, genome-wide association study; OR, odds ratio; PC, principal component; MDS, multidimensional scaling; MR-MEGA, meta-regression of multi-ethnic genetic association. Effect sizes are reported as odds ratios (OR) or β coefficients as described in the original studies.
